# Prostaglandin E1 Alleviates Cognitive Dysfunction in Chronic Cerebral Hypoperfusion Rats by Improving Hemodynamics

**DOI:** 10.3389/fnins.2019.00549

**Published:** 2019-05-29

**Authors:** Xiaomei Xie, Weibiao Lu, Yuanfang Chen, Chi Kwan Tsang, Jianye Liang, Wenxian Li, Zhen Jing, Yu Liao, Li’an Huang

**Affiliations:** ^1^Department of Neurology, The First Affiliated Hospital, Jinan University, Guangzhou, China; ^2^Clinical Neuroscience Institute, The First Affiliated Hospital, Jinan University, Guangzhou, China; ^3^Department of Radiology, The First Affiliated Hospital, Jinan University, Guangzhou, China; ^4^Department of Pathology, The First Affiliated Hospital, Jinan University, Guangzhou, China

**Keywords:** cerebral blood flow, chronic cerebral hypoperfusion, cognitive functions, neuronal damage, prostaglandin E1

## Abstract

Compensatory vascular mechanisms can restore cerebral blood flow (CBF) but fail to protect against chronic cerebral hypoperfusion (CCH)-mediated neuronal damage and cognitive impairment. Prostaglandin E1 (PGE1) is known as a vasodilator to protect against ischemic injury in animal models, but its protective role in CCH remains unclear. To determine the effect of PGE1 on cerebral hemodynamics and cognitive functions in CCH, bilateral common carotid artery occlusion (BCCAO) was used to mimic CCH in rats, which were subsequently intravenously injected with PGE1 daily for 2 weeks. Magnetic resonance imaging, immunofluorescence staining and Morris water maze (MWM) were used to evaluate CBF, angiogenesis, and cognitive functions, respectively. We found that PGE1 treatment significantly restored CBF by enhancing vertebral artery dilation. In addition, PGE1 treatment increased the number of microvascular endothelial cells and neuronal cells in the hippocampus, and decreased the numbers of astrocyte and apoptotic cells. In the MWM test, we further showed that the escape latency of CCH rats was significantly reduced after PGE1 treatment. Our results suggest that PGE1 ameliorates cognitive dysfunction in CCH rats by enhancing CBF recovery, sustaining angiogenesis, and reducing astrocyte activation and neuronal loss.

## Introduction

Chronic cerebral hypoperfusion (CCH) is a common consequence of various cerebral vascular disorders and hemodynamic changes, which commonly occurs in the elderly and diseases such as vascular dementia (VD) and Alzheimer’s disease (AD) (Shu et al., 2013; Zhao and Gong, 2015). CCH was known to cause aggravation of progressive cognitive deficits (Choi et al., 2015). It has been reported that CCH is linked to risk factors of VD and AD through chronic disruption of cerebral blood flow (CBF) resulting in neurological deficit and behavioral impairment (de la Torre, 2004; Oh et al., 2015). However, current therapeutic strategy in CCH research is mainly limited to neuroprotection (Anastacio et al., 2014; Gupta et al., 2015; Oh et al., 2015), whereas the use of other potentially important approach, such as revascularization during carotid occlusion, is still lacking (Maddula et al., 2017). Emerging evidence has shown that collateral circulation-enhancing strategies play critical role in restoring blood flow within ischemic regions. Because cerebral collateral circulation could provide the subsidiary network of vascular channels that stabilize CBF when principal conduits fail, adequate collateral circulation may contribute to the maintenance of tissue viability in the absence of re-canalization (Bang et al., 2015). Therefore, we hypothesize that a combinatorial therapy of restoring CBF and neuroprotection should be more desirable for CCH treatment.

The permanent rat model of bilateral common carotid artery occlusion (BCCAO) is the most commonly used model of CCH (Ji et al., 2010; Zhao and Gong, 2015). Our previous studies have shown that CBF decreases acutely after BCCAO and subsequently returns to the pre-occlusion level after 4 weeks of BCCAO. However, the restoration of CBF cannot ameliorate neurodegeneration and memory impairment (Jing et al., 2015). This may be related to the lack of timely neuroprotection after BCCAO. Currently, however, there is only a few effective interventions for CCH, including Dl-3-n-butylphthalide, human urinary kallidinogenase and statins (Xiong et al., 2017). Therefore, it is in urgent need to identify effective therapies to simultaneously accelerate CBF recovery and protect neurons in order to improve cognitive functions in patients with CCH.

Prostaglandin E1 (PGE1) is an E series prostaglandin which is a prostacyclin analog (Hafez et al., 2007). Previous studies of the pharmacological activities of PGE1 mainly focused on its effect on vasodilation. These studies showed that PGE1 can increase peripheral blood flow in heart, kidney, liver, and eyes in patients with peripheral vascular disease (Shin et al., 2012; Zhao et al., 2013; Yucel et al., 2015; Steigerwalt et al., 2016; Li et al., 2018). Emerging evidence has also shown that PGE1 activates angiogenesis and fibrinolysis, inhibits platelet aggregation, fibrinogenesis, inflammatory cell activity and proliferation (Schror and Hohlfeld, 2004; Huang et al., 2008; Gao et al., 2010). Recent studies further suggested that PGE1 can reduce infarct size and promote neurogenesis in acute ischemic stroke animal models (Sheng et al., 2011; Ling et al., 2016). However, the effect of PGE1 on hemodynamics and cognitive functions in chronic cerebral hypoperfusion or global cerebral ischemia remains unclear. This study comprehensively evaluated the efficacy of PGE1 in restoring CBF and ameliorating cognitive impairment in a rat model of chronic cerebral hypoperfusion.

## Materials and Methods

### Animals and Drug Administration

Adult male Sprague-Dawley rats (aged 3 months and weighing 300–380 g at the beginning of the study) were obtained from the Animal Experiment Center of Southern Medical University (Guangzhou, China). All animal procedures were performed in accordance with the recommendations in the Guide for the Care and Use of Laboratory Animals of the National Institutes of Health. The protocol was approved by the ethics committees at Jinan University. PGE1 was purchased from the Ted Pharmaceutical Co., Ltd., Beijing, China. PGE1 was dissolved in 0.9% saline (PGE1: 0.9% saline, 1:1) for intravenous injection. A total of 70 rats were randomly divided into seven groups as follows: two groups (the vehicle and PGE1-treated groups) were measured for CBF and vertebral artery (VA) diameters, and five groups [sham, Veh-2wk (vehicle-treated, observed at 2 weeks after BCCAO), PGE1-2wk (PGE1-treated, observed at 2 weeks after BCCAO), Veh-4wk (vehicle-treated, observed at 4 weeks after BCCAO), and PGE1-4wk (PGE1-treated, observed at 4 weeks after BCCAO) groups] were assigned for detection of behavior and pathology. PGE1-treated rats received daily tail-vein injection of PGE1 solution (6 μg/kg/day) and 9% saline for 14 days (from days 1 to 14 after BCCAO) (Hasegawa and Ichioka, 2015). Pathological changes in the rats were observed at 2 weeks (PGE1-2wk) and 4 weeks (PGE1-4wk) after BCCAO. The vehicle group received daily tail-vein injection of 9% saline at the same dose used for the PGE1- treated group for 14 days after BCCAO. Rats in the sham group underwent the same surgical procedures without BCCAO and received daily tail-vein injection of 9% saline at the same dose used for the PGE1- treated group for 14 days after BCCAO.

### Bilateral Common Carotid Artery Occlusion (BCCAO)

Bilateral common carotid artery occlusion (BCCAO) surgery was performed as described in our previous study (Jing et al., 2015; Xiong et al., 2017). CCH was induced by the modified permanent BCCAO method. Briefly, rats were initially occluded at the right common carotid artery (RCCA), and at 1 week after the initial occlusion, the rats were occluded at the left common carotid artery (LCCA). During the procedure, the rats were anesthetized with 3% pentobarbital sodium (0.1 ml/100 g) and the operating room was maintained at a temperature of 28.0 ± 2.0°C. Sham-operated rats were subjected to the same surgery without the occlusion of common carotid artery.

### Magnetic Resonance Imaging (MRI)

Magnetic resonance imaging (MRI) experiments were conducted with a Discovery 750 3.0T scanner with an 8-channel wrist coil (GE Healthcare, Milwaukee, WI, United States). The PGE1-treated and vehicle groups were scanned with MRI at six time points: pre-occlusion, 0-h-post-BCCAO, and 1, 2, 3, and 4 weeks after BCCAO. Following anesthesia with3% pentobarbital sodium (0.1 ml/100 g), animals were placed in a supine position prior to scanning. Detailed imaging parameters for the 3D ASL series are described in our previous study (Jing et al., 2015; Xiong et al., 2017).

### 3D Arterial Spin Labeling Technique (3D ASL)

Cerebral blood flow in different brain regions of rats was measured by the 3D Arterial Spin Labeling (ASL) technique. The detailed processes and parameters have been described in our previous study (Jing et al., 2015; Xiong et al., 2017).

### Morris Water Maze Task

Learning and spatial memory deficiencies caused by cerebral chronic hypoperfusion in the rats in each group were evaluated with the Morris water maze (MWM) test at 2 and 4 weeks after BCCAO, using a previously described method (Jing et al., 2015). For the experiment of navigation trials, all rats were subjected to 1 min of swimming adaptation training at 1 day before the trial, and the rats with poor swimming ability were eliminated. Next, the trial was conducted for 5 days at the same time each day. The rats were placed in a water-filled tank with four quadrants and one platform to swim for 60 s in each quadrant, with a 5-min interval between trial in each quadrant. The time required for a rat to find the platform and stay on the platform for more than 5 s was recorded by a video camera linked to an animal behavioral recording system (Ethovision XT; Noldus Information Technology Co., The Hague, Netherlands), and the time was called as escape latency. If a rat could not find the platform within 60 s, the latency would be recorded as 60 s, and the experimenter would guide it to the platform, let it stay on the platform for 10 s, and then put it back in the cage for 5 min before proceeding to the next test. The mean escape latency of four training sessions in a day was recorded as the daily learning score of an individual rat. For the experiment of probe trials, on the 6th day, the platform was removed, and the rats were placed in water at the 4th quadrant, which was the farthest from the original platform location. The swimming route and number of times that the rats crossed the platform in 60 s were recorded by the animal behavioral recording system.

### Tissue Preparation

At 2 and 4 weeks after BCCAO, rats (*n* = 5 in each group) were anesthetized as described above and transcardially perfused with physiologic saline followed by 4% paraformaldehyde in 0.1 mol/L phosphate-buffered saline (PBS, pH 7.4). The brain was dissected immediately and fixed in 4% paraformaldehyde 12 h at 4°C. Coronal blocks from the optic chiasm to the posterior level of hypothalamus, which includes the hippocampus and parietal cortex (PC), were prepared and then processed for dehydration with an increasing alcohol gradient and three xylene exposures in an Automated Tissue Processor (LYNX II, Hatfield, PA, United States). The blocks were embedded with paraffin in an embedding machine (HistoStar; Thermo Scientific, Kalamazoo, MI, United States). Coronal sections were cut at of 10-mm thickness with a paraffin microtome (RM2235; Leica, Wetzelar, Germany).

### Immunofluorescent Labeling

Immunofluorescence analysis was performed as previously described (Jing et al., 2015; Xiong et al., 2017). Briefly, sections were processed for immunofluorescent labeling with CD34 antibody (for endothelial cells of microvessels), glial fibrillary acidic protein (GFAP) antibody (for astrocytes), NeuN antibody (for neurons) and cleaved caspase-3 (C-caspase 3) antibody (for apoptotic cells). The sections were immersed in blocking solution (5% normal goat serum or donkey serum in PBS) at 20°C for 2 h, then incubated 12 h at 4°C with CD34 antibody (rabbit, 1:100; Abcam, United States), GFAP antibody (rabbit, 1:1000; Abcam, United States),and NeuN antibody (mouse, 1:100; Abcam, United States) and cleaved caspase-3 antibody (rabbit, 1:100; Abcam, United States). After three times of washing with 0.01 M PBS, the sections were incubated with secondary antibody (Alexa Fluor 488-conjugated goat anti-rabbit IgG, 1:400; Jackson Immunoresearch, West Grove, PA, United States). CY3- conjugated goat anti-rabbit or goat anti-mouse IgG, 1:300; Servicebio,China) for 2 h at 20°C. After three times of washing in PBS, the sections were covered with anti-quenching fluorescence mounting medium.

### Detection of Cerebral Blood Flow (CBF)

3D-ASL images with a scanner software (Functool 3D ASL, v4.5, GE Medical Systems, Milwaukee, WI, United States) were used to calculate CBF. Regions of approximately 2 mm × 2 mm from the parietal cortex, basal ganglion and hippocampal regions were selected for CBF measurement. A total of 48 regions from these regions in each group were analyzed at each time point.

### Measurement of Vertebral Arteries (VAs)

Vertebral arteries were captured by 3D TOF angiography. The diameter of the left and right VAs were measured at the middle of the cervical region. A total of 16 diameter values in each group were analyzed at each time point.

### Analysis of Numbers of CD34-, GFAP-, NeuN-, and C-Caspase-3- Positive Cells

For pathological detection, eight rats in each group were used. In each group, a total of 32 sections at 100 μm intervals for each time point were selected for immunofluorescent labeling. Digital images were captured under 400× magnification from the CA1 and CA3 subfields of the hippocampus and PC. A total of 32 photos were taken from both side of the CA1 region, CA3 region, or PC region in each group at one time point separately. The numbers of CD34-, GFAP-, NeuN-, and C-caspase-3- positive cells in each image were counted with ImagePro Plus version 6.0 software (Media Cybernetics Co., United States). Measurements were performed by two individuals blind to the study parameters.

### Analysis of Spatial Memory Function

Spatial memory function was analyzed from the escape latency and frequency of rats crossing the original platform. Data were transferred from the animal behavioral recording system to the SPSS version 24 software (SPSS IBM, New York, NY, United States) for analysis. At least 32 numerical values in each group at different time points were analyzed.

### Statistical Analysis

All data were tested for normal distribution using the One-sample Kolmogorov–Smirnov test with SPSS version 24 (SPSS IBM). Normally distributed data were presented as mean ± standard deviation (SD). For the data that were not normally distributed, they were expressed as median ± interquartile range. The repeated measures of analysis of variance (ANOVA) of the general linear model and the LSD *post hoc* test were conducted in SPSS to compare different measured times of CBF. An uncorrected *p*-value of <0.05 was used to ensure the maximum time of CBF restoration and the significance of the findings. Similarly, the diameter of VAs was analyzed by repeated measurement with Bonferroni *post hoc* test to compare different measure time. Multivariate ANOVA was used to compare CBF and diameter of VAs between the vehicle and PGE1 groups at the six time points. Second, the data of escape latency from the MWM test were transferred to SPSS and analyzed by repeated measurement and multivariate ANOVA with Tukey *post hoc* test (if equal variances were assumed) or Tamhane’s T2 test (if equal variances were not assumed). Finally, data on the numbers of CD34-, GFAP-, NeuN-, caspase-3- positive cells, as well as data on the frequency in the platform quadrant from the MWM test, were analyzed by one-way ANOVA with Tukey *post hoc* test (if equal variances were assumed) or Tamhane’s T2 test (if equal variances were not assumed). The charts were drawn by GraphPad Prism 6.0 software (GraphPad Software, La Jolla, CA, United States). Statistical significance was set at *p* < 0.05.

## Results

### PGE1 Promoted CBF Recovery in the Parietal Cortex, Hippocampus, and Striatum After BCCAO

**FIGURE 1:**
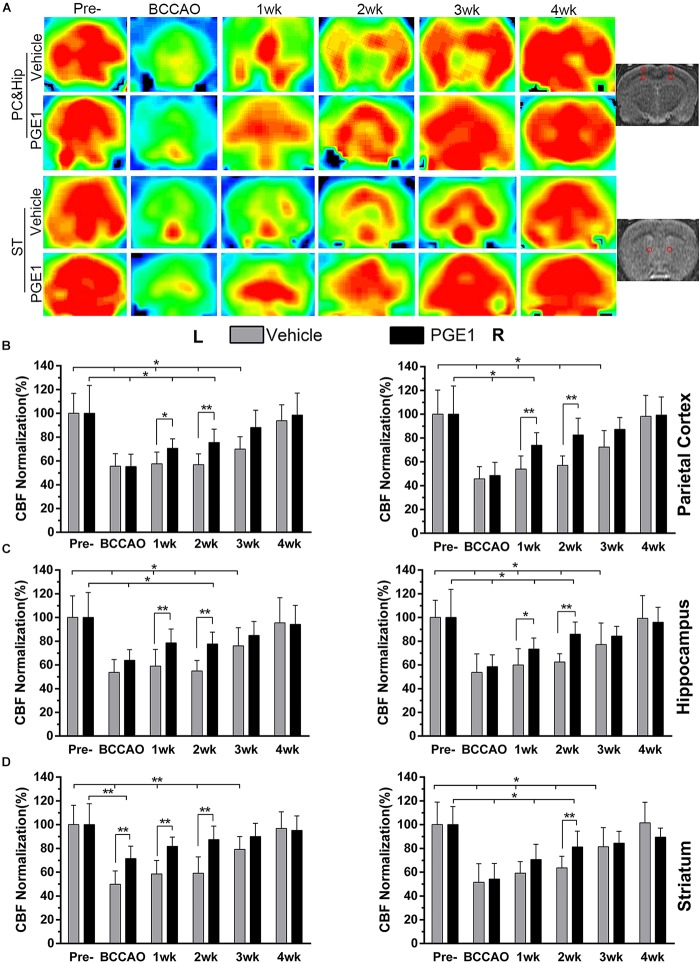
PGE1 treatment accelerates CBF recovery in BCCAO rat model. **(A)** MRI 3D ASL analysis was used to monitor changes in CBF in the cortex, hippocampus (rows 1, 2), and striatum (rows 3, 4) of the vehicle- (rows 1, 3) and PGE1-treated (rows 2, 4) rat brains at the indicated time points following BCCAO. Images at the right column indicate the corresponding brain regions. The red dots in the right image represent the range and location of the measurement. The red color in the heat map represents increased CBF level, whereas green indicates lower CBF level. CBF quantitative analysis results in the parietal cortex **(B)**, hippocampus **(C)**, and striatum **(D)** in the vehicle- and PGE1-treated groups at the indicated time points after BCCAO. Data are presented as mean ± SD; *n* = 8; ^∗∗^*p* < 0.01, ^∗^*p* < 0.05. PC, parietal cortex; Hip, hippocampus; ST, striatum; Pre, pre-occlusion; BCCAO, bilateral common carotid artery occlusion; CBF, cerebral blood flow; L, left; R, right.

We measured CBF with 3D Arterial Spin Labeling Technique in the parietal cortex (PC), hippocampus and striatum of rats in the vehicle and PGE1-treated groups at the time of pre-occlusion, beginning of BCCAO, and 1–4 weeks after BCCAO. Color signals from green to red represent an increasing gradient of CBF level (Figure 1A). Consistent with our previous observation (Jing et al., 2015; Xiong et al., 2017), red signal appeared in the brain areas including the cortex, hippocampus and striatum in both groups at the pre-occlusion time (Figure 1A). After BCCAO, CBF in the vehicle-treated rats decreased, as judged by the shifting of signal to the green end of the spectrum. The low CBF persisted until 3 weeks post-occlusion when red signals reappeared in the cortex, hippocampus and striatum, and then reached pre-occlusion levels at 4 weeks after BCCAO (Figure 1A). In contrast to the vehicle control, the PGE1-treated animals recovered at a faster rate (Figure 1A). Quantitative analysis indicated that in the right cortex, CBF in vehicle-treated rats was dramatically reduced to 45.64% ± 10.27 upon BCCAO (*p* < 0.01, 95% CI: pre-occlusion, 83.28–116.72%; post-BCCAO, 37.53–53.75%), and then moderately returned to the normal level at 4 weeks post-BCCAO (*p* > 0.05, 95% CI: 85.63–110.69%, Figure 1B). In the PGE1-treated group, however, CBF at 1 week after BCCAO was significantly higher than that in vehicle group (*p* < 0.01, 95% CI: PGE1-treated group, 65.73–82.13%; vehicle-treated group, 45.69–62.09%). In addition, the level of CBF returned to the normal level after 2 weeks of BCCAO (*p* > 0.05, 95% CI: 73.79–91.20%, Figure 1B). The pattern of changes in CBF and the effect of PGE1 on the left cortex were similar to those of the right cortex (Figure 1B).

With respect to the hippocampus, CBF in the left side also markedly decreased to 53.77% ± 10.86 during the period of BCCAO in the vehicle group (*p* < 0.01, 95% CI: pre-occlusion, 85.07–114.93%; post-BCCAO, 46.20–61.33%), and then gradually recovered to 95.45% ± 21.27 at 4 weeks after BCCAO (*p* > 0.05, 95% CI: 81.19–109.72%). Treatment with PGE1 particularly enhanced CBF in both sides of the hippocampus at 1 week post-BCCAO (*p* < 0.01, 95% CI: PGE1-treated group, left side, 68.73–88.36%; *p* < 0.05, 95% CI: PGE1-treated group, right side, 64.35–82.22%, respectively, Figure 1C). Remarkably, PGE1 treatment caused CBF recovery to the normal level in both side of the hippocampus at 3 weeks after BCCAO. In the left side of the striatum, PGE-1 treatment also enhanced CBF at 1 week post-BCCAO (*p* > 0.05, 95% CI: 74.45–89.09%, Figure 1D). These results show that PGE1 promoted the recovery of CBF after BCCAO.

### PGE1 Ameliorated Learning and Memory Impairments After BCCAO

**FIGURE 2:**
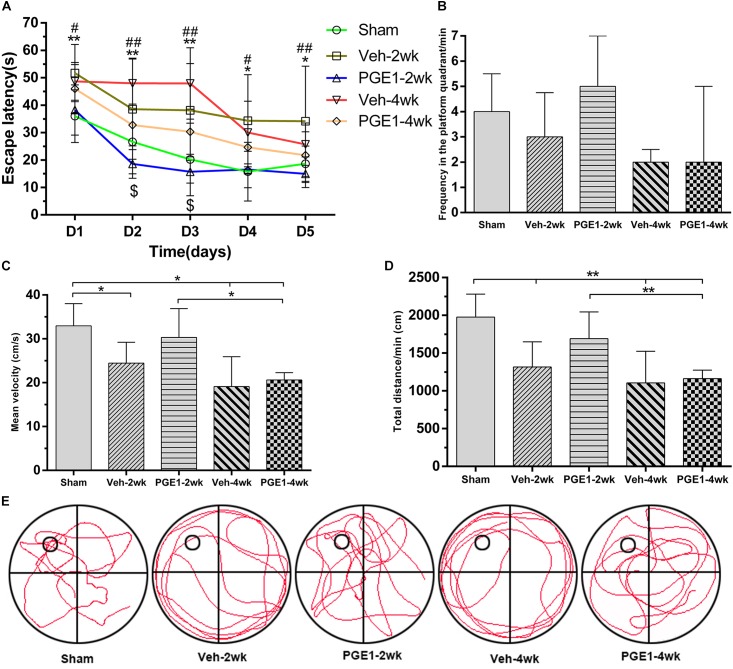
PGE1 alleviates cognitive impairment as measured by Morris water maze (MWM) test. **(A)** Escape latency changes in the different groups from days 1 to 5 of MWM test. Data are presented as mean ± SD; *n* = 10, 8, 10, 9, 9, respectively. (^∗∗^*p* < 0.01, ^∗^*p* < 0.05, sham group vs. Veh-2wk or Veh-4wk groups; ^##^*p* < 0.01, ^#^*p* < 0.05, PGE1-2wk group vs. Veh-2wk group; ^$^*p* < 0.05, PGE1-4wk group vs. Veh-4wk group). **(B)** Changes between different groups in the frequency of time in the platform quadrant in 1 min. Data are presented as median ± interquartile range; *n* = 10, 8, 10, 9, 9, respectively. **(C)** Quantitative analysis of the mean velocity in 1 min at day 6 of MWM test between the different groups. Data are presented as mean ± SD; *n* = 10, 8, 10, 9, 9, respectively. **(D)** Quantitative analysis of the total distance in 1 min between the different groups. Data are presented as mean ± SD; *n* = 10, 8, 10, 9, 9, respectively. **(E)** Swimming path of rats in different groups at day 6 of MWM test. The small empty circle represents the platform in one quadrant of the swimming pool (the large circle). Rats of the Veh-2wk and Veh-4wk groups were mainly swimming along the side wall, whereas the rats of the sham, PGE1-2wk and PGE1-4wk groups were searching for the platform purposefully.

To investigate whether the observed enhancement of CBF recovery by PGE1 could improve cognitive functions in CCH, we examined the learning and memory functions by analyzing escape latency and frequency in the platform quadrant using MWM test. As shown in Figure 2A, the escape latency of the vehicle-treated rats from days 1 to 5 of MWM test was significantly longer than that of the sham group at 2 and 4 weeks after BCCAO (all *p* < 0.05, e.g., 95% CI at day 1: Sham group, 30.23–41.79 s; Veh-2wk group, 45.24–58.15 s; Veh-4wk group, 42.58–54.75 s), validating the establishment of learning and memory deficits in our BCCAO model. Remarkably, PGE1-treated animals at 2 weeks after BCCAO showed dramatically reduced escape latency from days 1 to 5 of MWM test, compared to the vehicle-treated group (all *p* < 0.05, e.g., 95% CI at day 1: Veh-2wk group, 45.24–58.15 s; PGE1-2wk group, 32.46–44.01 s). At 4 weeks after BCCAO, the escape latency of PGE1-treated rats was significantly reduced at day 2 and day 3 of MWM test, compared to that of the vehicle-treated group (*p* < 0.05, e.g., 95% CI at day 2: Veh-4wk group, 40.58–55.36 s; PGE1-4wk group, 25.37–40.16 s, Figure 2A). With respect to the frequency of time in the platform quadrant, PGE1-treated group also showed an increased frequency compared with that of the vehicle group, although the differences were not statistically significant (Figure 2B). However, the mean velocity and total distance at day 6 showed the differences between these groups. As shown in Figure 2C, the mean velocity of the sham group was higher than that of the Veh-2wk, Veh-4wk, and PGE1-4wk groups (all *p* < 0.05, 95% CI: Sham group, 29.31–36.57 cm/s; Veh-2wk group, 20.44–28.44 cm/s; Veh-4wk group, 13.91–24.33 cm/s; PGE1-4wk, 19.34–21.91 cm/s), except the PGE1-2wk group (*p* > 0.05, 95% CI: PGE1-2wk group, 25.60–35.60 cm/s). In contrast, the mean velocity of the PGE1-2wk group was higher than that of the PGE1-4wk (*p* < 0.05), which indicated that PGE1 treatment was effective in alleviating the learning and memory impairment caused by CCH. Result of the total distance measurement showed similar trend to that of mean velocity (Figure 2D). We also performed analysis of the swimming path at day 6 of MWM test. As shown in Figure 2E, rats in the Veh-2wk and Veh-4wk groups showed a more monotonous paths, whereas PGE1-treated rats swam in a more versatile paths which were similar to those of the sham group (Figure 2E). These results show that PGE1 mitigated the learning and memory deficits caused by BCCAO.

### PGE1 Stimulated Dilation of Bilateral Vertebral Arteries After BCCAO

**FIGURE 3:**
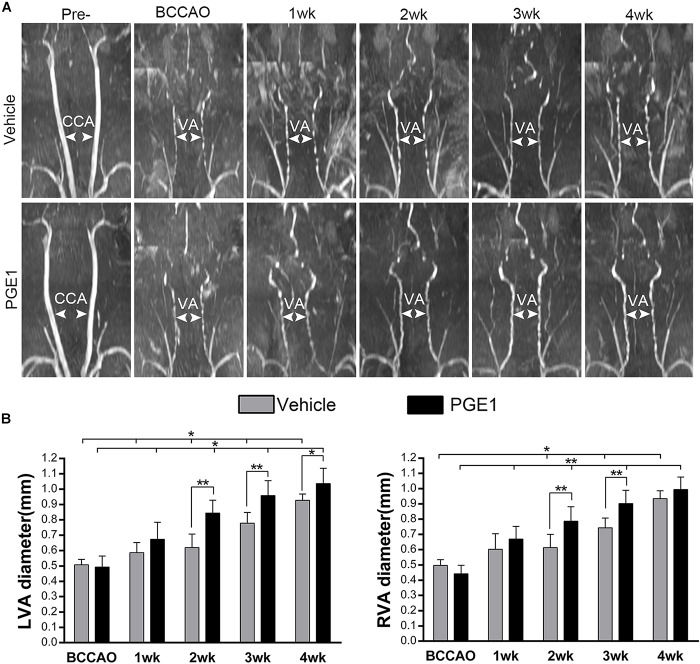
PGE1 treatment promotes vasodilation in the vertebral arteries. **(A)** MRI 3D-TOF angiography was used to capture the morphological changes and diameter of the vertebral arteries (VAs) in the vehicle- and PGE1-treated groups following BCCAO. Common carotid arteries (CCAs) and VAs were indicated by white arrowheads. **(B)** Quantitative analysis showing changes in left (L) and right (R) VA diameter from 1 to 4 weeks in the vehicle- and PGE1-treated groups. Data are presented as mean ± SD; *n* = 8. ^∗∗^*p* < 0.01, ^∗^*p* < 0.05.

Because PGE1 is a well-known potent vasodilator (Hasegawa and Ichioka, 2015; Lee, 2017). To investigate whether the enhancement of CBF and cognitive functions by PGE1 was attributed to vascular alteration, we examined the effect of PGE1 on vertebral arteries (VAs). As shown in Figure 3A, the VAs in both pre-occlusion groups were undetectable owing to their narrow size. However, VA diameter in the vehicle group gradually increased after BCCAO, which is consistent with our previous finding (Jing et al., 2015). Treatment with PGE-1 significantly enhanced VA diameter, compared to treatment with vehicle after 2 weeks of BCCAO (Figure 3A). Quantitative analysis results are shown in Figure 3B. The diameter of left VAs in the vehicle-treated rats increased from 0.51 mm ± 0.03 at the beginning of BCCAO to 0.93 mm ± 0.04 at 4 weeks after BCCAO (*p* < 0.01, 95% CI: post-BCCAO: 0.48–0.54 mm, 4 weeks after BCCAO: 0.89–0.96 mm). In PGE1-treated group, the diameter of left VAs was significantly larger than that in the vehicle group at 2 weeks (*p* < 0.01, 0.85 mm ± 0.08 vs. 0.62 mm ± 0.08, 95% CI: 0.78–0.92 mm, 0.55–0.69 mm, respectively), 3 weeks (*p* < 0.01, 0.96 mm ± 0.09 vs. 0.78 mm ± 0.07, 95% CI: 0.88–1.04 mm, 0.72–0.84 mm, respectively) and 4 weeks (*p* < 0.05, 1.04 mm ± 0.10 vs. 0.93 mm ± 0.04 95% CI: 0.95–1.12 mm, 0.89–0.96 mm, respectively) post-BCCAO (Figure 3B). The effect of PGE1 on the right VAs was similar to that on the left side (Figure 3B). These results suggest that PGE1 promoted vasodilation and growth of VAs in the BCCAO rats.

### PGE1 Sustained Angiogenesis in the Cortex and Hippocampus After BCCAO

**FIGURE 4:**
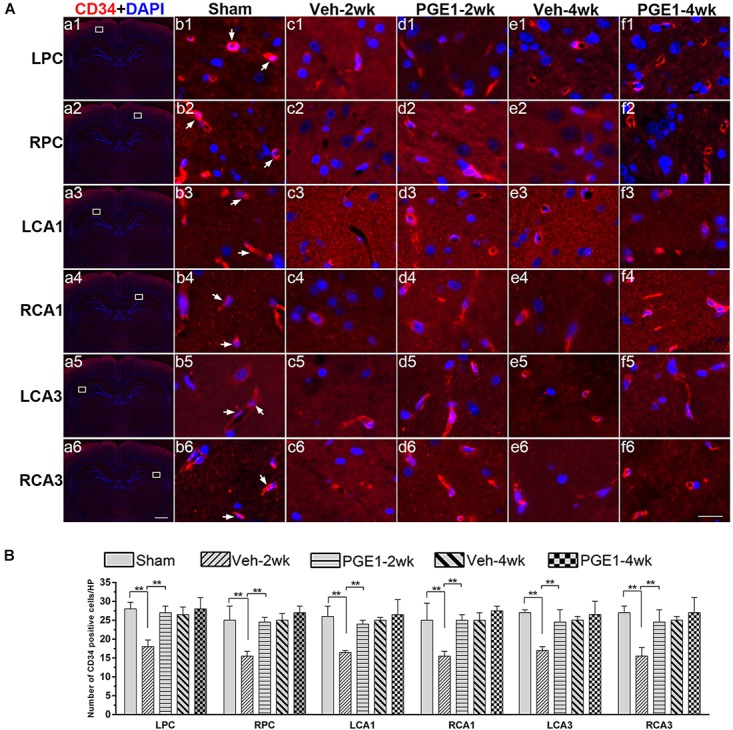
Changes in angiogenesis of immunolabeled microvessels in response to vehicle and PGE1 treatment. **(A)** Immunofluorescent staining of CD34-positive cells in the microvessels of the left parietal cortex (LPC), right parietal cortex (RPC), left CA1 area (LCA1), right CA1 area (RCA1), left CA3 area (LCA3), and right CA3 area (RCA3) in the sham, vehicle group at 2 weeks after BCCAO (Veh-2wk), vehicle group at 4 weeks after BCCAO (Veh-4wk), PGE1-treated group at 2 weeks after BCCAO (PGE1-2wk) and PGE1-treated group at 4 weeks after BCCAO (PGE1-4wk). Microphotographs in column 1 (a1–a6) show the sections stained with fluorescent double-staining of CD34 (red) and DAPI (blue). The white squares in the microphotographs show the selected position. Microphotographs in column 2 to 6 (b1–f6) show parts of the magnified view of specific areas. CD34 positive cells were indicated by white arrowheads. Scale bar: a1–a6, 1,000 μm; b1–f6, 20 μm. **(B)** Quantification of CD34-positive cell density in the indicated brain regions at different time points after BCCAO. Data are presented as median ± interquartile range; *n* = 8; ^∗∗^*p* < 0.01. CD34, cluster of differentiation 34.

To further explore the accelerating effect of PGE1 on CBF recovery, we investigated whether PGE1 promoted angiogenesis after BCCAO. We employed immunofluorescence staining of CD34, an endothelial progenitor cell marker (Siemerink et al., 2012; Fan et al., 2018), to detect changes in angiogenesis in the PC and the CA1 and CA3 regions of the hippocampus at 2 and 4 weeks after BCCAO (Figure 4A). In these three regions, BCCAO-induced hypoperfusion was associated with a significant decrease in the number of CD34-positive cells at week 2 after BCCAO (all *p* < 0.01, e.g., 95% CI at LPC: Sham group, 26.48–30.02; Veh-2wk group, 16.09–19.41). The number of CD34-positive cells returned to the normal level at week 4 after BCCAO (all *p* > 0.05, e.g., 95% CI at LPC: Sham group, 26.48–30.02; Veh-4wk group, 24.95–28.05, Figure 4B). Strikingly, PGE1 treatment alleviated the decrease of CD34-positive cells at 2 weeks after BCCAO (all *p* > 0.05, e.g., 95% CI at LPC: Sham group, 26.48–30.02; PGE1-2wk group, 24.58–28.67). At 4 weeks, the numbers of CD34-positive cells in the PGE1-treated group were even higher than those in the vehicle-treated group, although there was no statistically significant difference. These findings showed that PGE1 may reduce the loss of endothelial progenitor cells and sustain angiogenesis after BCCAO.

### PGE1 Suppressed Astrocyte Activation in the Hippocampus After BCCAO

**FIGURE 5:**
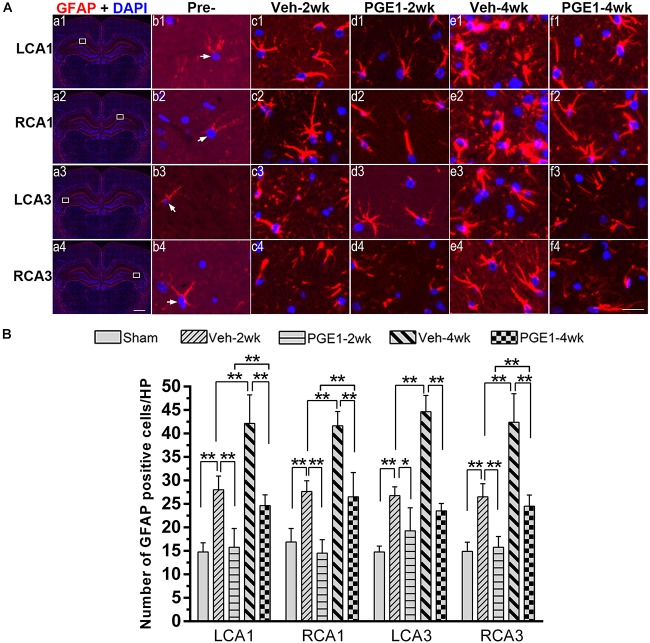
Changes in astrocyte activation in response to vehicle and PGE1 treatment. **(A)** Astrocyte was stained by immunofluorescent labeling with GFAP antibody (red) and DAPI (blue) in LCA1, RCA1, LCA3, and RCA3 in the sham, Veh-2wk, Veh-4wk, PGE1-2wk, and PGE1-4wk groups. GFAP positive cells were indicated by white arrowheads. Scale bar: a1–a4: 1,000 μm; b1–f4: 20 μm. **(B)** Changes in astrocyte density, as measured by the number of GFAP-positive cells/HP in these regions at different times following BCCAO. Data are presented as mean ± SD; *n* = 8; ^∗∗^*p* < 0.01, ^∗^*p* < 0.05. GFAP, glial fibrillary acidic protein.

In our previous study (Jing et al., 2015), we found that the number of activated astrocytes increases owing to prolongation of the CCH process, resulting in progressive cognitive impairment. We reasoned that PGE1 ameliorated learning and memory impairments, as shown in Figure 2, by reducing astrocyte activation. To test this hypothesis, we investigated the effect of PGE1 on astrocyte activation by GFAP immunostaining in the hippocampus. Temporal changes in GFAP immunofluorescent labeling in each group are shown in Figure 5A. Quantitative analysis results showed that in the vehicle group at 2 weeks after BCCAO, the densities of GFAP-positive cells in LCA1 and RCA1 increased compared with those in the sham group (all *p* < 0.01, e.g., 95% CI at LCA1: Sham group, 13.09–16.41; Veh-2wk group, 25.55–30.45, Figure 5B). The cell densities were further increased at 4 weeks after BCCAO (all *p* < 0.01, 95% CI at Veh-4wk groups: LCA1, 37.02–47.23; RCA1, 39.06–44.19). PGE1 treatment reduced the increased GFAP-positive cells at 2 and 4 weeks following BCCAO (all *p* < 0.01; Veh-2wk vs. PGE1-2wk, e.g., 95% CI at LCA1: 25.55–30.45, 12.38–19.12, respectively; Veh-4wk vs. PGE1-4wk, e.g., 95% CI at LCA1: 37.02–47.23, 22.68–26.57, respectively). In the CA3 area, the number of GFAP-positive cells and temporal pattern of change in both sides were similar to those in the CA1 region (Figure 5B). These findings suggested that PGE1 could suppress astrocyte activation.

### PGE1 Reduced Neuronal Loss and Apoptosis in the Hippocampus After BCCAO

**FIGURE 6:**
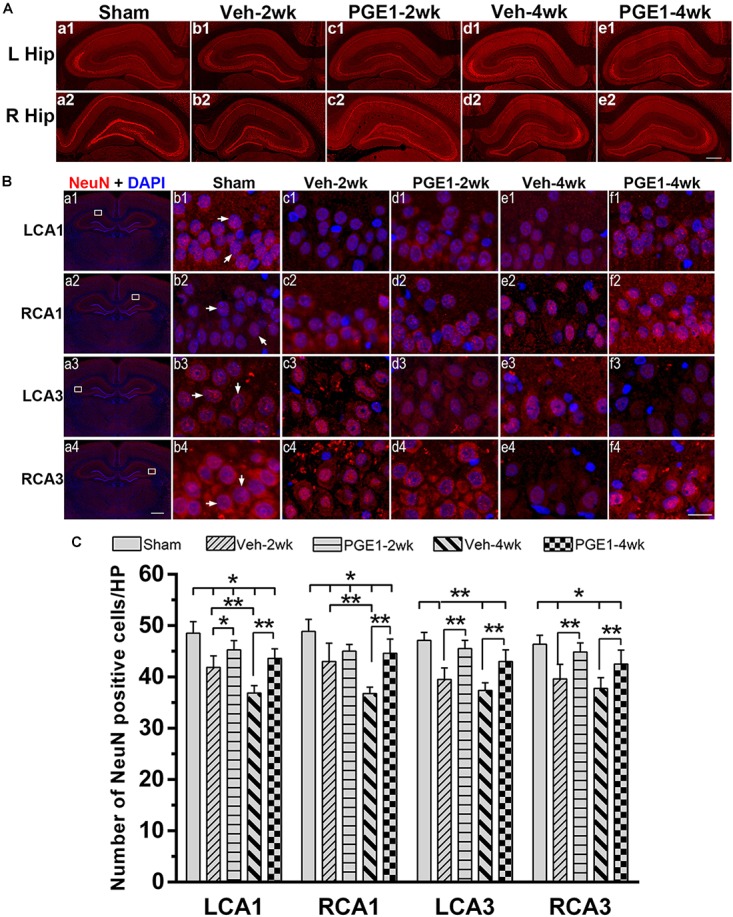
PGE1 treatment mitigates neuronal loss after BCCAO. **(A)** Representative overview pictures of hippocampus of NeuN staining. Scale bar: 500 μm. **(B)** Neuronal cell density was determined by immunostaining with NeuN antibody (red). The nuclei were stained with DAPI (blue). Representative images of NeuN positive cells in the LCA1, RCA1, LCA3, and RCA3 areas in the sham, Veh-2wk, Veh-4wk, PGE1-2wk, and PGE1-4wk groups. NeuN positive cells were indicated by white arrowheads. Scale bar: a1–a4: 1,000 μm; b1–f4: 20 μm. **(C)** Changes in neuron density in these areas at different times following BCCAO. Data are presented as mean ± SD; *n* = 8; ^∗∗^*p* < 0.01, ^∗^*p* < 0.05. NeuN, neuron-specific nuclear-binding protein.

**FIGURE 7:**
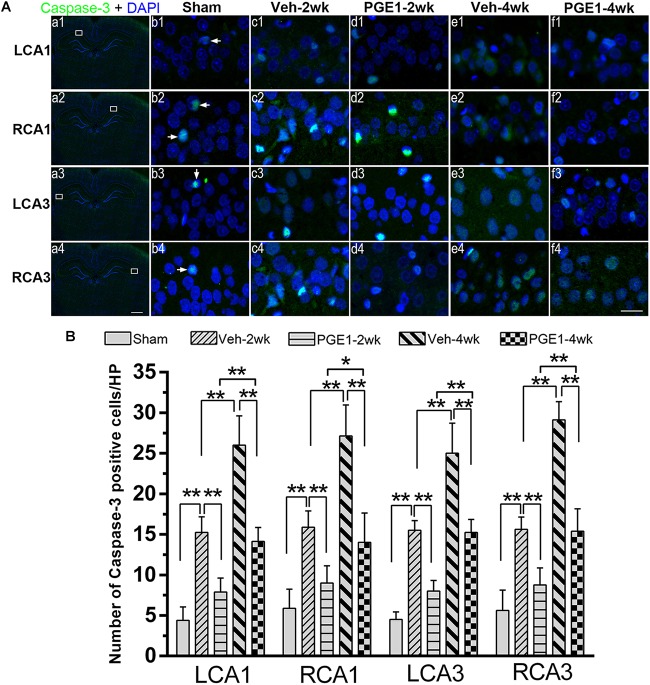
PGE1 treatment reduces apoptosis induced by BCCAO. **(A)** Apoptotic cells were stained by immunofluorescent labeling using cleaved caspase-3 antibody (red). The nuclei were stained with DAPI (blue). Representative images of cleaved caspase-3 positive cells in the LCA1, RCA1, LCA3, and RCA3 areas in the sham, Veh-2wk, Veh-4wk, PGE1-2wk, and PGE1-4wk groups. Cleaved caspase-3 positive cells were indicated by white arrowheads. Scale bar: a1–a4: 1,000 μm; b1–f4: 20 μm. **(B)** Changes in the number of caspase-3 positive cells in the LCA1, RCA1, LCA3, and RCA3 regions at different time points after BCCAO. Data are presented as mean ± SD; *n* = 8; ^∗∗^*p* < 0.01, ^∗^*p* < 0.05. Caspase-3, cysteinyl aspartate specific proteinase 3.

We further determined the number of neurons in the hippocampal CA1 and CA3 regions by NeuN immunostaining. Figure 6A showed the overview pictures of hippocampus with NeuN staining. The number of neurons in sham group was higher than other groups and PGE1-treated groups were higher than the vehicle-treated groups. As shown in Figure 6B, BCCAO resulted in significant reduction in the number of NeuN-positive cells in all analyzed hippocampal regions in the vehicle-treated rats at 2 and 4 weeks after BCCAO. PGE1 treatment significantly reduced the loss of NeuN-positive cells at 2 and 4 weeks after BCCAO (all *p* < 0.05; Veh-2wk vs. PGE1-2wk, e.g., 95% CI at LCA1: 40.01–43.74, 43.72–46.78, respectively; Veh-4wk vs. PGE1-4wk, e.g., 95% CI at LCA1: 35.66–38.09, 42.08–45.17, respectively). In addition, this PGE1-mediated effect was also observed in both sides of the hippocampus (Figure 6C). To verify that the loss of neurons was due to apoptosis, we used cleaved-Caspase-3 immunostaining to label apoptotic cells in the CA1 and CA3 regions (Figure 7A). Quantitative analysis indicated that the number of caspase-3- positive cells markedly increased in all analyzed hippocampal regions at 2 weeks after BCCAO (all *p* < 0.05, Sham vs. Veh-2wk/PGE1-2wk, e.g., 95% CI at LCA1: 2.97–5.78, 13.65–16.85, 6.43–9.32, respectively). The number of caspase-3- positive cells further increased at 4 weeks after BCCAO (all *p* < 0.05, Sham vs. Veh-4wk/PGE1-4wk, e.g., 95% CI at LCA1: 2.97–5.78, 22.97–29.03, 12.68–15.57, respectively, Figure 7B). However, PGE1 treatment significantly alleviated the increase in the number of caspase-3 positive cells in all hippocampal regions at 2 and 4 weeks after BCCAO (all *p* < 0.01, Veh-2wk vs. PGE1-2wk, Veh-4wk vs. PGE1-4wk, e.g., 95% CI at LCA1 were shown above, Figure 7B). These results suggest that PGE1 reduces neuronal loss and apoptosis in the hippocampus after BCCAO.

## Discussion

In the present study, we discovered that PGE1 treatment significantly alleviates cognitive impairment in cerebral hypoperfusion in BCCAO model rats. We found that this novel therapeutic effect of PGE1 is apparently associated with its accelerating effect of CBF restoration. We further revealed that PGE1-mediated CBF enhancement is associated with dilation of VAs and sustaining angiogenesis. Furthermore, we showed that PGE1 reduces neuronal loss and apoptosis, as well as astroglial activation, probably through PGE1-mediated CBF enhancement. According to our knowledge, this is the first report relating PGE1-mediated CBF enhancement to amelioration of cognition deficits in CCH. This study provided a preclinical evidence suggesting that PGE1 has promising potential to be developed as an effective therapeutic drug to treat CCH.

To comprehensively assess hemodynamics in CCH model rats, we used 3D ASL, 3D TOF and immunofluorescence staining to measure CBF, diameter of VAs, and CD34-positive cells, respectively. 3D ASL is commonly used to measure CBF because it facilitates non-invasive and repeatable examinations without the use of contrast agents (Nery et al., 2018; Hu et al., 2019). Using this method, we found that PGE1 promotes CBF recovery in the parietal cortex, hippocampus and striatum after BCCAO. Our result is consistent with the finding by Hosoi’s group, which suggests that PGE1 can be used as a cerebral vasodilator (Yamanaka et al., 2014). In addition, it has been shown that PGE1 therapy for patients with hypertensive intracerebral hemorrhage after stroke is capable of enhancing regional CBF of perihematomal tissue (Cao et al., 2011). However, it has been reported that PGE1 does not influence cerebrocortical blood flow, as measured by Laser-Doppler flowmeter, in acute middle cerebral artery occlusion ischemic animal models (Han et al., 2008; Sheng et al., 2011). This discrepancy may be due to the different ischemic models and experimental settings used in these studies. It is important to elucidate the underlying mechanism of PGE1 for vasodilation in future studies.

By 3D TOF analysis, we found that PGE1 acts on VAs and improves blood flow in the brain of animal CCH model. However, we cannot rule out the possibility that PGE1 also acts on other cerebral vessels for its effect on improving CBF and cognitive functions. It has been proposed that PGE1 may improve symptoms in patients with lumbar spinal canal stenosis by promoting blood flow in the cauda equina and nerve roots through its vasodilating and anti-platelet aggregating effects (Yoshihara, 2016). In addition, Miyabe et al. (2001) showed that cerebral microvessel diameter in rabbits is increased by intravenous administration of PGE1. As our previous finding has shown that the VAs have the ability to increase their diameter as an adaptive response to BCCAO (Jing et al., 2015). The effect of PGE1 on enhancing CBF observed in the present study may be contributed, at least in part, to its vasodilating action on the VAs.

In addition, we further revealed that PGE1 sustains angiogenesis, thereby promoting CBF restoration in CCH. Consistent with our finding, it has been reported that PGE1 promotes angiogenesis in ischemic limb in patients with diabetes by potentiating the impaired angiogenic properties of bFGF (Huang et al., 2008). PGE1 can also up-regulate the expression of VEGF in ischemic stroke model rats and vascular cognitive impairment in rats, which may be mediated by lipo-PGE1 via the VEGF/VEGFR pathway (Ling et al., 2016, Liu et al., 2017). Considering that PGE1 has multiple pharmacological effects (Tonseth et al., 2017), further investigation is required to clarify the correlation between the effect of PGE1 on angiogenesis and CBF in CCH.

Evidence from animal experiments and clinical trials has suggested that the early and marked CBF restoration in ischemic brain plays a critical role in tissue repair and functional recovery after ischemic injury (Li et al., 2007; El Amki and Wegener, 2017). Accumulating evidence has shown that adaptive responses in the brain following CCH include angiogenesis and dilation of collateral vessels for CBF restoration (Jing et al., 2015; Nishijima et al., 2016; Xiong et al., 2017). In this study, we found that astrocyte activation, neuronal loss and apoptosis may also contribute to cognitive impairment in CCH rats. As PGE1 could accelerate CBF restoration, it may alleviate these effects in CCH rats.

Astrocytes play critical roles in coupling neuronal activity to CBF, and modulating excitatory synaptic transmission (Maragakis and Rothstein, 2006). Following injury in the central nervous system, astrocytes become reactivated and change their morphology. This phenomenon is known as astrogliosis, which is characterized by rapid synthesis of GFAP (Eng et al., 2000; Yang and Wang, 2015). In this study, we showed that the number of GFAP-positive cells in the hippocampus of CCH rats is significantly increased, whereas PGE1 can alleviate astrocyte activation. After the interruption of PGE1 administration, the number of GFAP-positive cells in the PGE1-treated group increased dramatically. These results strongly suggest that PGE1 inhibits astroglial activation. Currently, the underlying mechanism remains unknown. Since it has been reported that PGE1 might improve ipsilateral reperfusion and decrease the number of reactive astrocytes and lesion volume in neonatal stroke model rats (Bonnin et al., 2018), we speculate that PGE1-mediated CBF enhancement may contribute to the reduction in astrocyte activation, which in turn may reduce inflammatory responses and alleviate cognitive impairment. In addition, it has been reported that endoplasmic reticulum stress plays critical roles in determining the degree of astroglial activation after brain ischemia (Yoshikawa et al., 2015). Future research is required to elucidate the regulation of PGE1-mediated reduction in astroglial activation in CCH.

Neuron damage is typical in brain ischemia. Our results also showed that rat CCH causes reduction in neuronal numbers, and PGE1 alleviates neuronal loss. We further found that the numbers of Caspase-3- positive cells in the PGE-treated groups were lower than those in the vehicle groups, indicating that PGE1 treatment contributes to the reduction in apoptosis in CCH. Considering our finding that PGE1 accelerates CBF restoration and relieves hypoperfusion, we reason that PGE1-mediated CBF enhancement may contribute to the reduction in neuronal loss and apoptosis. PGE1-mediated reduction in neuronal loss and apoptosis may consequently ameliorate learning and memory deficits in CCH. Future research is required to elucidate the mechanism of the ameliorative effects of PGE1 on neuronal loss and apoptosis.

## Ethics Statement

This study was carried out in accordance with the recommendations of guidelines from the Institutional Review Board of the First Affiliated Hospital of Jinan University. The protocol was approved by the Institutional Review Board of the First Affiliated Hospital of Jinan University.

## Author Contributions

XX and WLu performed all experiments, performed data analyses, and wrote the manuscript. YC performed the MWM tests and performed data analyses. CT wrote and edited the manuscript. JL provided technical input in the MRI process. WLi, ZJ, and YL performed the pathological experiments. LH contributed in the conception and design of the study, as well as wrote and edited the manuscript.

## Conflict of Interest Statement

The authors declare that the research was conducted in the absence of any commercial or financial relationships that could be construed as a potential conflict of interest.
